# Sea-Sickness

**Published:** 1872-07

**Authors:** 


### SEA-SICKNESS.

Since it has become so fashionable to cross the Atlantic for a tour on the continent, and, as almost every one suffers more or less from sea sickness, any suggestions that would lead to an amelioration of the distressing symptoms accompanying this sickness, if not to entirely prevent it, would be hailed with more than ordinary joy by the migratory public.

Sea-sickness has been attributed to so very many different causes, that it would be difficult to enumerate them, much less to explain the arguments of their various projectors. It has been attributed to fear, nervousness, or mere imagination; but, while there are instances where it could be traced to such sources, it is by no means so in the great majority of people who are victims to it.

Dr. Wallaston, an eminent English surgeon, who wrote upon this subject years ago, claimed that the sickness was produced by pressure of blood upon the brain; since which time his views have been supported by the pathological observations of other eminent physicians, who have shown by a variety of experiments, that pressure on the brain is always accompanied by sickness of the stomach and consequent vomiting. Dr. Wallaston explains the manner in which pressure is produced upon the brain during the motion of a ship or a swing, by reference to the action of the mercury in a barometer. He says: "If a barometer be carried out to sea in a calm, the mercury will rest at the same height as when on shore; but when the ship falls by the subsidence of the waves, the mercury is seen to rise in the tube." This fact is more forcibly illustrated, however, in the ordinary carrying of a barometer; when, if you happen to let it descend suddenly, by lowering the hand holding it, you will soon be apprised of the consequent rising of the mercury in the tube, by hearing and feeling its dull thug against the top of the tube. Now, the action of the blood upon the brain, when the ship is descending from the top of a wave, is identical with that of the mercury in the tube, which is lowered by the hand; and in the more violent descents of the ship, the pressure upon the brain, by the rushing of the blood from the extremities, amounts to an actual blow; this, when frequently repeated, produces nausea and consequent vomiting.

The same flow of blood to the brain occurs in the ordinary amusement of swinging, which process is often quite as nauseating as the motion of a vessel. In the act of swinging, the dizziness and nausea occurs when you are descending forwards, so that the blood by its own inertia, comes in contact with the brain, while the body with its numerous vessels are moving forward and downward.

Sea-sickness, then, is produced first, by pressure upon the brain; secondly, on the stomach through the sympathetic nerves; and thirdly, through the imagination.

The means, therefore, to avoid this train of causes are first, to lie down; this is generally done, however, without being told; but to lie down in the proper position requires some reasoning. You doubtless have observed that the sickness always occurs, not when the ship is rising on the wave, but when it is descending from it, producing a feeling as though all support is taken from under you. The reason being, as before explained, that the blood is forced against the brain, or more correctly speaking, the brain is thrown against the blood, producing congestion and shock. In the rising of the ship, the reverse occurs, and the brain is relieved of its superabundance of blood, and the pressure is renmoved, until the next descent of the craft. Now, if taking your recumbent position, you are careful to lie with your head toward the bow of the boat, the blood will be driven in the direction of your feet instead of the head, so that no congestion will ensue from the forward and downward pitch of the vessel. Still better, if you can secure your state room near the middle of the ship, and arrange your cot so that your head and feet are pointed in the direction of the sides of the vessel, no congestion of the brain can arise when the vessel is riding the waves.

But, if from any cause, the vessel rocks laterally like a cradle, your position must be changed to accommodate the new motion.

Therefore, if you are careful to observe the following rules upon entering a vessel for a journey upon the water, we firmly believe that you will not be disturbed by sea sickness: Be sure that your stomach contains a full, wholesome meal; secure your stateroom near the center of the ship, if possible; arrange your bed crosswise of the vessel; lie down before the vessel starts, and remain lying for the first twenty-four hours; take six drops of chloroform in a little water, should you feel nausea coming on; this will produce a pleasant warmth of the stomach and arrest the nausea; go upon deck when the weather is pleasant, and spend the most of your time there; find something to occupy your mind or fingers; go to your meals regularly, and resort to the lying-down and chloroform process should you feel a disposition to sickness.

And now you have our theory and cure for sea sickness.

